# Why Drafted Dental Students Should Be Permitted to Finish Their Courses

**Published:** 1917-09

**Authors:** 


### WHY DRAFTED DENTAL STUDENTS SHOULD BE PERMITTED TO FINISH THEIR COURSES

*This data has been gathered for the information of those interested in the maintenance of ample dental service for the United States Army.*

A few weeks after the United States joined the Allies in the war against Germany it was announced on seemingly good authority in Washington *that it was the desire of the Surgeon General's Office and the War Department that all medical and dental students should continue their studies and that they certainly would not be called into service until after graduation.* It was apparently appreciated that there would be an ever-increasing demand for more and more physicians and dentists, and as no one could foretell the duration of the war, it seemed that the production of physicians and dentists should not be reduced. The United States was to profit by the example of England, France and Canada and would not make the mistake of sending partly trained medical men to the front in the line service. The announcement became common knowledge throughout the country and such sensible action was generally accepted as right.

It was not until after the registration for the draft that the War Department apparently became frightened over the problem of class exemptions, and announced that no exemptions would be made except those provided by law. Pressure was immediately brought by the medical profession, and after weeks of argument, an order was issued by General Crowder on August 29, 1917, that hospital internes and fourth, third and second year medical students who were called into service might enlist in the Enlisted Reserve Corps (Regulations for the Enlisted Reserve Corps, Act of June 3, 1916) and permitted to complete their course. *This act does not grant exemption. It holds the men in the Reserve Corps, subject to call.*

It was generally presumed that the dental service was considered a valuable and necessary part of the medical service of the army and that dental students would be included in such an order. It was therefore a surprise to both physicians and dentists throughout the country that dental students were omitted. While practically every argument which has been presented favoring the temporary exemption of medical students applies to dental students as well, the following information has been gathered by the Committee of Deans of the dental colleges of the United States for those who may be interested, as applying more especially to dental students.

### NO LEGAL OBSTACLE TO ISSUANCE OF DENTAL ORDER

There is no legal obstacle to the issuance of an order for dental students, similar to that issued for medical students. The regulation specifies that "Engineer, Signal and Quartermasters Corps and the Ordnance and Medical Departments of the Regular Army" may have such Enlisted Reserve Corps. As the army dental service is one of the medical departments, it might be included in such an order. *Attention is called to the fact that the issuance of such an order would not "let down the bars" to an endless line of similar orders, as the regulations referred to apply to but the three corps and two departments above mentioned.*

### EFFECT OF DRAFT ON DENTAL STUDENTS

At the request of this committee the deans of a majority of the dental colleges throughout the country sent blanks to all underclassmen of last year to be filled in and returned direct to the chairman of this committee. Each dean was also requested to report the number of those in attendance in each class last year and to give best estimate of the number who will matriculate in the freshman class this fall.

From the blanks returned the following table has been prepared:

| | |
|---|---|
| Total number of underclassmen in dental schools last year | 7624 |
| Number to whom blanks were mailed | 6510 |
| Number of blanks returned to date | 3935 |
| Number not of draft age, or alien | 1120 |
| Number in draft | 2815 |
| In first call | 697 |
| Accepted | 517 |
| Rejected | 181 |
| Percentage of all students called in first draft | 17.7 |
| Percentage of students of draft age who were called in first draft | 24.7 |
| Percentage of all students accepted in first call | 13.1 |
| Percentage of drafted students accepted in first call | 18.3 |
| Percentage of all students who have volunteered in army or navy | 5.0 |
| Total number of students of last year who will be accepted through the draft at above percentage rate | 1395 |
| Total number of students of last year who have volunteered | 381 |
| Total number entering army through first draft or as volunteers | 1776 |
| Total freshmen matriculated in all schools last year | 4184 |
| Estimate of freshmen this year | 1330 |

From the above it will be noticed that a total of 1776 students have already been taken from the classes which will graduate in 1918 and 1919. In accordance with the blanks returned, a few more will be included in the second call than the first. Allowing for errors in the calculations of some students, it is safe to estimate that the second call will include as many as the first. Several schools report having already received many letters from this group stating that they will not return, as they do not wish to go to the expense in time and money with the chances so greatly against their being permitted to complete another year's work. Even presuming that the next draft does not come until late next spring (so that those who do attend might receive credit for the year's work), it seems a fair estimate that at least 5 per cent., in addition to those drafted and volunteered, of the graduating class of 1918 will not return this fall because of the second call, and that the full 13.1 per cent. for the second call may properly be deducted from the class of 1919, as they will not reach graduation. It is to be presumed that at least a third draft will also be made before this class graduates, making a similar further reduction. We must also deduct an additional 10 per cent. from the class of 1918, and at least 15 per cent. from the class of 1919 to cover the normal loss in these classes from many causes—sickness, faculty action, financial difficulties, etc.

*On these calculations, the effect of the draft, plus the normal loss, will be to take in the neighborhood of 966 students from the class of 1918, and 2271 students from the class of 1919.*

Owing to the fact that all dental schools have extended their courses from three to four years, beginning this fall, there will be no graduating classes in 1920.

It will be noted that the class of 1921 (freshman class of this fall) will be about one-third of last year's class, this reduction being undoubtedly due in large measure to the draft.

### SUPPLY OF DENTAL STUDENTS ALREADY LESS THAN DEMAND

On account of the development of the important relationship of mouth infections to general health, and the education of our people to the value of healthy mouths, the demand for dental service has increased much faster in recent years than the supply of dentists. Therefore, under the most favorable conditions, it would be impossible for the dental schools of the country to turn out during the next five years as many dentists as will be needed. If the army takes all it needs, the civilian population must suffer. It therefore seems almost imperative that the greatest possible number of dentists should be graduated.

The number of graduates from all of the dental schools of the United States for the past five years was as follows:

| | |
|---|---|
| 1913 | 2022 |
| 1914 | 2254 |
| 1915 | 2388 |
| 1916 | 2835 |
| 1917 | 2989 |
| Total | 12488 |

This is an average of 2,497 per year.

The best estimate that can now be made for the next five years is as follows:

| | |
|---|---|
| 1918 | 2474 |
| 1919 | 1913 |
| 1920 | practically none |
| 1921 | 1000 |
| 1922 | 800 |
| Total | 6187 |

This is an average of 1237 per year.

*It is thus apparent that the effect of the draft will be to reduce the number of graduates for the next five years to about one-half the number graduated during the past five years, and it will necessarily be four years after the close of the war before the graduating classes will begin to increase again in numbers.*

### PRESENT RATIO OF ARMY DENTISTS INADEQUATE

The following is quoted from an editorial in the September *Dental Cosmos:* "The government has provided for a corps of army and navy dental surgeons in the ratio of one dental surgeon to every one thousand enlisted men. Carefully computed statistical data tend to show that the average available working time of a dentist is about two thousand hours per year, upon the basis of which it appears that the government has made provision for an average of one hour of dental service every six months for each enlisted man—an amount of time wholly inadequate for the work required to be done. Any dentist who is giving full and efficient service to five hundred patients per year is in full practice. No dentist can give proper service in accordance with modern standards of practice to one thousand patients per year. To attempt to serve that number under army conditions tends to lower the standard of the service itself and is demoralizing to those who are engaged therein.''

Every dentist who has studied this problem knows that there should be at least one dentist for each 500 men in the army; just double the present number of dentists. A bill is now being prepared for presentation in Congress which provides for this increase. Obviously there should be foresight to have ready not only the one to one thousand now provided for, but also the more adequate service which will very likely be provided for by law.

### DENTAL PROFESSION HAS SHOWN FINE PATRIOTISM

In spite of the fact that the Washington authorities have shown little tendency to recognize the value of the services of the dental profession, its members have exhibited as fine a spirit of patriotism as any class of citizens possibly could. Realizing the very bad condition of the mouths of many conscripts and the inadequacy of the government service, the dental schools throughout the country have thrown open wide the doors of their clinic rooms and have, without charge to men or government, cared for the mouths of both conscripts and those who were refused enlistment on account of the conditions of their teeth. There has also been organized the Preparedness League of American Dentists, consisting of thousands of dentists throughout the country, and these men have, in private offices and elsewhere, given their services to the army men. *By this gratuitous service the dental profession has made possible the enlistment of several times the number of men (who would otherwise have been rejected) that will be called from the dental schools by the draft.*

It should also be mentioned that large numbers of dental students offered to enlist in the various base hospital units and other branches of army and navy service, and they were almost universally advised that they would render the most patriotic service by remaining in school. Many were thus deprived of entering officers' training camps and branches of service for which they had some special qualification.

General Crowder recently wrote to the governor of New York a letter strongly commending the work of dentists in the army, and said there was great need for a larger proportion of dentists.

*Attention is called to the fact that the members of the class of 1918 will be able to finish their dental training and be ready to enter the army dental service at as early a date as their training could be completed for line service, and the class of 1919 could be made ready for graduation by January 1 of that year.* All dental schools will modify their courses to give special training for army service, so that the graduates of next year will be in some ways better qualified than dentists who have been in regular practice for some years.

In presenting this statement, it hardly seems necessary that we should pledge the fullest possible degree of loyalty of the dental profession and its schools to the government. We believe it our highest duty to see to it, if we can, that the dental needs of the army and of the civilian population be cared for in the best manner possible. This is our aim.

Attention is called to the following affidavit stating the effect of three years of war on the dental service of Canada:

*Dominion of Canada, Province of Ontario, County of York,*
*To wit:*

I, A. E. Webster, M.D., D.D.S., L.D.S., Dean of the Royal College of Dental Surgeons of Ontario, of the City of Toronto in the County of York, do solemnly declare, that,

1. Three years of war under the voluntary system of recruiting has reduced the number of dentists in practice in Canada by one-fifth. The dental colleges with all possible effort have not been able to supply the demands of civil practice. Dental assistants for the army are supplied from the physically fit dental students while the unfit graduate to supply the places of those who have left civil practice to join the army.

2. Dentistry is an admitted necessity, especially in the army.

3. It is my opinion, based upon experience that a larger than normal supply of dental students should be admitted to the dental colleges to supply dental assistants for the army and graduates to take the places in civil practice of experienced dentists who take up military practice. And I make this solemn declaration, conscientiously believing it to be true and knowing that it is of the same force and effect as if made under oath and by virtue of "The Canada Evidence Act."

Declared before me at the City of Toronto, in the County of York, this 31st day of July, A. D. 1917.

A. E. Webster, M.D., D.D.S., L.D.S.

E. T. Coatsworth,

A Notary Public in and for the Province of Ontario.
[Seal.]

All of which is respectfully submitted.

Henry W. Morgan, Nashville, Tenn.; H. E. Friesell, Pittsburgh, Pa.; W. H. G. Logan, Chicago, Ill.; F. D. Casto, Cleveland, Ohio; Arthur D. Black, Chicago, Ill., Chairman, 122 So. Michigan Ave., Committee of Deans of Dental Colleges.

